# Cancer in adolescents and young adults in Japan: epidemiology and cancer strategy

**DOI:** 10.1007/s10147-021-02064-x

**Published:** 2021-11-15

**Authors:** Kayo Nakata, Eiso Hiyama, Kota Katanoda, Tomohiro Matsuda, Yuma Tada, Masami Inoue, Keisei Kawa, Mitsue Maru, Chikako Shimizu, Keizo Horibe, Isao Miyashiro

**Affiliations:** 1 Cancer Control Center, Osaka International Cancer Institute, 3-1-69 Otemae, Chuo-ku, Osaka, 541-8567 Japan; 2 Natural Science Center for Basic Research and Development, Hiroshima University, Hiroshima, Japan; 3 Division of Surveillance and Policy Evaluation, National Cancer Center Institute for Cancer Control, Tokyo, Japan; 4 Division of International Health Policy Research, National Cancer Center Institute for Cancer Control, Tokyo, Japan; 5 Department of Hematology, Osaka International Cancer Institute, Osaka, Japan; 6 Department of Hematology/Oncology, Osaka Women’s and Children’s Hospital, Osaka, Japan; 7 College of Nursing Art and Science, University of Hyogo, Hyogo, Japan; 8 Department of Breast and Medical Oncology, National Center for Global Health and Medicine, Tokyo, Japan; 9 Department of Pediatrics, National Hospital Organization Nagoya Medical Center, Nagoya, Japan

**Keywords:** Adolescent and young adult (AYA), Cancer, Epidemiology, Cancer strategy, Cancer care system

## Abstract

According to national cancer registry data in Japan, approximately 20,000 adolescents and young adults (AYAs, age 15–39 years) are newly diagnosed with cancer each year. Improvements in treatment and care for AYAs with cancer are included in the Phase Three Basic Plan to Promote Cancer Control Programs in Japan. This article reviews current cancer incidence and survival for AYAs with cancer in Japan using population-based cancer registry data. Mortality data through 2019 from the Vital Statistics of Japan are also described. Encouragingly, the 5-year survival probability for AYA cancers has continued to improve, in parallel with childhood cancers, and the mortality rate has decreased. There has been increasing attention to these vulnerable patients and improved partnerships and collaboration between adult and pediatric oncology; however, obstacles to the care of this population still exist at multiple levels. These obstacles relate to specific areas: research efforts and enrollment in clinical trials on AYA malignancies, AYA-specific psychosocial support such as education, financial support, and oncofertility care, and cancer care systems. It is important for Japanese oncologists, health care providers, and health policy makers to recognize that the AYA population remains vulnerable and still have unmet needs.

## Introduction

According to national cancer registry data in Japan, approximately 20,000 adolescents and young adults (AYAs, age 15–39 years) are newly diagnosed with cancer each year [1]. A previous report found that this age group has not shown the same improved survival as either older or younger cohorts [2]. There are many unique aspects of care to consider in this population that may influence outcomes both during and after therapy [2]. These include developmental status of the age group, psychosocial difficulties, barriers to access to specialized centers, a lack of specialist care guidelines and clinical trials relevant to AYAs, and differences in cancer biology and chemotherapy pharmacokinetics in cancer types [2–9]. These issues may complicate medical care and mean that AYAs require additional support compared with either older adults or younger children [10].

The National Cancer Control Act was established in Japan in 2006, initially focusing on major adult cancers [11]. In 2018, improvements in treatment and care for AYAs with cancer were introduced in the Phase Three Basic Plan to Promote Cancer Control Programs in Japan [12].

In this article, we present data on cancer incidence, survival, and mortality among AYAs with cancer in Japan based on population-based cancer registry data or Japanese demographic statistics. The discussion focuses on access to clinical trials, age-appropriate psychosocial support such as education, financial support and oncofertility care, and current cancer care systems for AYAs in Japan. Finally, we discuss the future of AYA oncology care in Japan.

## AYA cancer epidemiology in Japan

### Materials and methods

Cancer incidence data (2016–2018) to calculate AYA case distribution were obtained by applying for use of the population-based National Cancer Registry in Japan which was established in 2016. Recent population-based survival data (2009–2011) were obtained in the framework of the Monitoring of Cancer Incidence in Japan (MCIJ) project which includes data from 22 prefectures that follow-up on vital status of patients [13, 14]. Information on trends in survival (1975–2011) was obtained from the Osaka Cancer Registry [15]. For incidence and survival, invasive cancer cases, as defined by the International Classification of Diseases, 10th revision (ICD-10 codes, C00-96), were included. All cases were classified using the International Classification of Diseases for Oncology, third edition [16] and grouped according to the Surveillance, Epidemiology, and End Results (SEER) AYA definitions [17], which use both topographical site and histology information. For cancer mortality, we obtained the annual number of cancer deaths and age-standardized mortality rates with ICD-10 codes (C00-97) from published Vital Statistics of Japan [18] and the graph database of Cancer Statistics in Japan [19].

### Cancer occurrence in AYAs

**Fig. 1:**
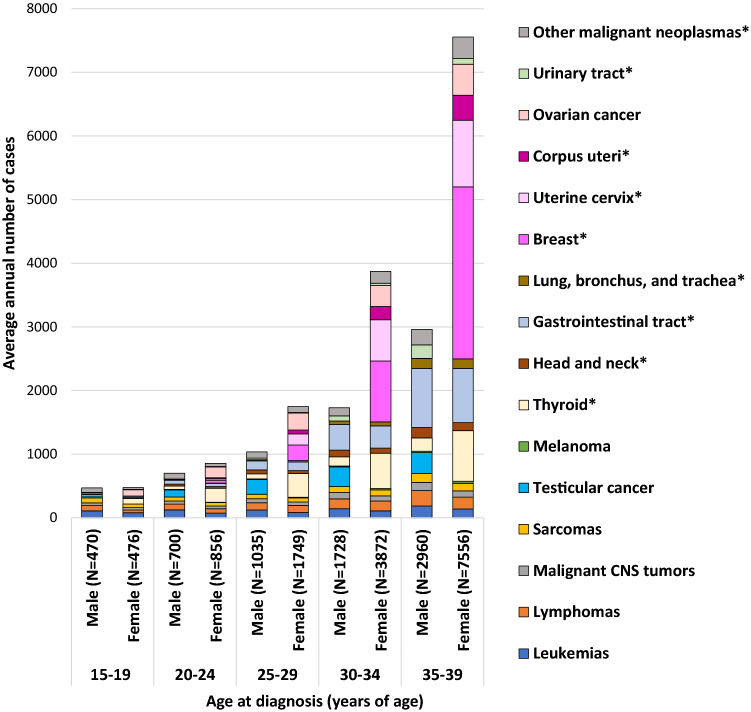
Average annual number of cases of selected adolescent and young adult cancer types by age group, 2016–2018. Excludes benign and borderline brain tumors. Coding for these cancers is based on the SEER AYA Site Recode 2020 Revision. CNS indicates central nervous system. N represents the average number of cases per year (2016–2018) of all cancer types in each age group. *Indicates carcinomas. Source: National Cancer Registry in Japan

According to national cancer registry data, AYA cancers accounted for 2% of all newly diagnosed invasive cancers in Japan between 2016 and 2018 [1]. Age-standardized incidence rates using world standard population [20] were 53.3 in both sexes, 35.1 in males, and 72.1 in females per 100,000 person years (2016–2018). Among this generation, females have a greater incidence rate than males. The distribution of cancer types varies across AYA age groups and sexes (Fig. 1). For example, hematologic malignancies are the most common cancers in adolescents (age 15–19 years) in both sexes, with leukemias and lymphomas accounting for 40% in males and 26% in females. In addition, sarcomas and malignant central nervous system (CNS) tumors account for a larger percentage of all cancers in the group aged 15–19 years (sarcomas: 17% in males, 11% in females, malignant CNS tumors: 10% in males, 8% in females), relative to other age groups. Testicular cancer was the most common cancer among males aged 25–29 years (22%), while gastrointestinal carcinoma was the most common cancer among males aged 30–34 years (23%) and 35–39 years (31%). Thyroid carcinoma and ovarian cancer were the most common cancers among females aged 20–24 years (thyroid: 26%, ovary: 20%) and 25–29 years (thyroid: 21%, ovary: 15%), while carcinomas of the breast and uterine cervix were the most common cancers among females aged 30–34 years (breast: 25%, uterine cervix: 17%) and 35–39 years (breast: 36%, uterine cervix: 14%).

### Trends in AYA cancer survival and mortality

**Fig. 2:**
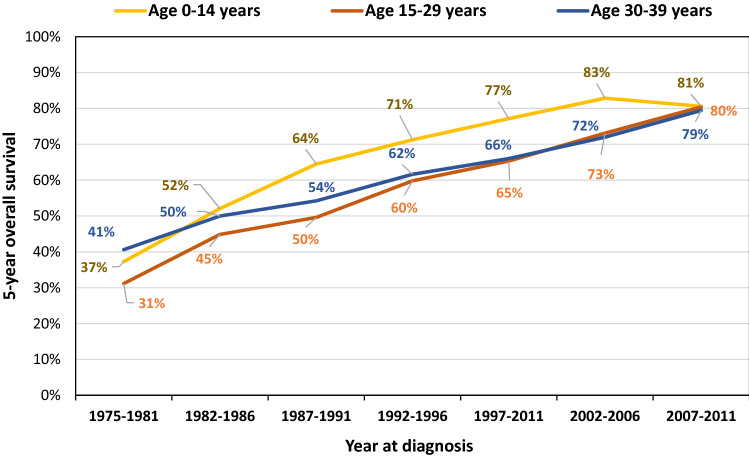
Trends in 5-year overall survival from cancer among children, adolescents, and young adults, 1975–2011. Adapted from: Cancer Control Center, Osaka International Cancer Institute. “Incidence and survival of cancer in children, adolescents, and young adults in Osaka.” https://osaka-gan-joho.net/link/childhood-cancer/pdf/2020_pdf_cc3.pdf#page=2. Source: Osaka Cancer Registry

**Fig. 3:**
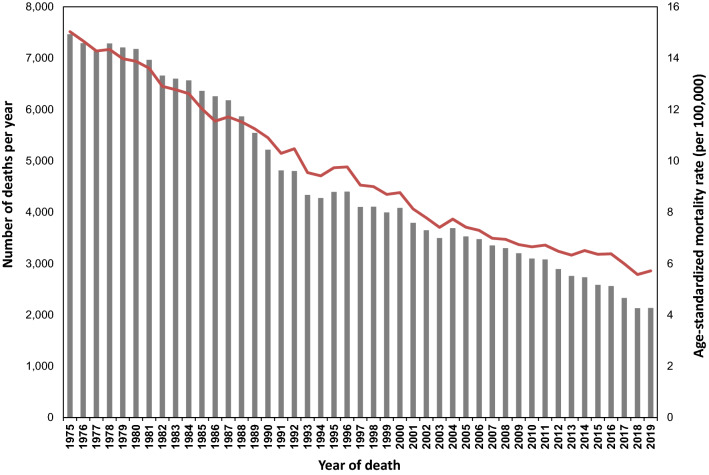
Trends in cancer mortality among adolescents and young adults (age 15–39 years), 1975–2019. World standard population was used for age standardization. Source: Vital Statistics of Japan

According to trends in population-based cancer survival data from the Osaka Cancer Registry, 5-year overall survival among AYAs for all cancers increased from 31% (age 15–29 years) and 41% (age 30–39 years) for patients diagnosed in the mid-1970s to approximately 80% for those diagnosed during 2007 through 2011 (Fig. 2). From the late 1980s to the early 2000s, the 5-year overall survival for AYAs was about 10% lower than for children (age 0–14 years), but survival for AYAs has improved and by the late 2000s was similar to that for children (Fig. 2). Within the AYA population, survival varies by cancer type or age subgroup (15–29 years and 30–39 years) (Table 1). For example, the 5-year survival for patients diagnosed with thyroid carcinoma, testicular cancer, and corpus uteri carcinoma during 2009 through 2011 was over 90% in both age groups, but only 53.0% (age 15–29 years) and 44.7% (age 30–39 years) for patients with carcinoma of the lung, bronchus, and trachea (Table 1). The 5-year survival of patients aged 15–29 years with sarcomas, carcinomas of the gastrointestinal tract, breast, and urinary tract was lower than that of patients aged 30–39 years by over 5% (Table 1). Since the 1970s, the mortality rate of the AYA generation has decreased significantly (Fig. 3). However, cancer remains the leading disease-related cause of death in this generation, with more than 2000 adolescents and young adults dying from cancer each year even recently in Japan (Fig. 3) [18]. By cancer type, leukemia was the most common cause of death among males and females aged 15–29 years, while colorectal cancer was the most common among males aged 30–39 years, and breast cancer among females aged 30–39 years in 2019 [18].

**Table 1:** Five-year overall survival for leading adolescent and young adult cancer types, 2009–2011

	Age 15–29 years			Age 30–39 years		
	5-year OS (%)	95% CI		5-year OS (%)	95% CI	
Leukemias	74.7	70.8	78.1	77.1	73.5	80.2
Lymphomas	88.8	85.4	91.5	87.7	84.9	90.1
Malignant CNS tumors	64.2	57.5	70.0	62.2	56.5	67.4
Sarcomas	70.9	65.6	75.6	78.8	74.5	82.5
Testicular cancer	95.1	91.8	97.1	96.9	95.2	98.0
Ovarian cancer	93.8	89.9	96.2	79.6	75.6	83.0
Melanoma	92.2	77.6	97.4	74.8	62.2	83.8
Thyroid^1^	99.8	98.4	100.0	99.3	98.6	99.7
Head and neck^1^	87.2	80.2	91.9	85.2	81.4	88.2
Gastrointestinal tract^1^	59.1	53.6	64.3	66.7	64.7	68.6
Lung, bronchus, and trachea^1^	53.0	40.1	64.4	44.7	39.9	49.4
Breast^1^	84.8	79.8	88.7	90.6	89.5	91.6
Uterine cervix^1^	90.1	86.3	92.9	88.5	86.9	89.9
Corpus uteri^1^	92.2	82.2	96.7	91.0	87.8	93.3
Urinary tract^1^	81.5	68.3	89.6	87.3	83.1	90.6

Excludes benign, borderline, in situ neoplasms

*CI* confidence interval, *CNS* central nervous system, *OS* overall survival probability

^1^Indicates carcinomas, Source: Monitoring of Cancer Incidence in Japan (MCIJ) project

## Challenging and improving outcomes of AYAs with cancer

Despite improving survival trends, AYAs with cancer continue to experience inferior outcomes compared with younger and older cohorts in several cancer types [21, 22]. Certainly, unique genetic and biologic features explain some of the differences [23–25]. AYAs with cancer have different support needs from other age groups in dealing with new cancer diagnoses as well as ongoing long-term and late effects of treatment [26, 27]. These issues can be addressed by increasing research efforts and enrollment in clinical trials targeting AYA cancers, expanding access to care, and providing AYA-specific psychosocial support [10].

### Access to clinical trials

It is well documented that enrollment into clinical trials is fundamental to improving clinical outcomes for cancer patients [28]. However, enrollment in the AYA age group was reported to be inferior to pediatric and adult participation [28–32]. To reduce these age disparities and increase clinical trial enrollment, national organizations and cooperative trial groups have made efforts to bridge the age gaps in research protocols [10, 28]. In the USA, the Southwest Oncology Group (SWOG) and the Children’s Oncology Group (COG) have specific committees dedicated to the AYA population [10]. Many of the COG research protocols for leukemia and sarcomas extend the eligibility age to ≥ 30 years [10]. In Europe, the European Society for Medical Oncology (ESMO) and the European Society for Paediatric Oncology (SIOPE) established the joint Cancer in AYA Working Group. In Japan, although reports on the participation rate in clinical studies are scarce, it has been reported that clinical trial enrollment was 23% for AYAs with acute lymphoblastic leukemia (ALL) aged 15–29 years diagnosed during 2001–2005 in Osaka [6]. In the field of hematological oncology, the Japan Adult Leukemia Study Group (JALSG) and Japan Children’s Cancer Group (JCCG) have jointly set up clinical studies for AYA patients; for example, the upper age limit for clinical studies of ALL in children has been raised to 64 years. In addition, the Japan Society of Clinical Oncology (JSCO), a leading Japanese professional organization founded in 1963, established the AYA Cancer Treatment Review Committee in 2018. It is hoped that these organizations will take the lead in monitoring clinical research participation or in considering appropriate clinical approaches for the AYA generation in each cancer type.

### Psychosocial factors and support

In Japan, the legal age of independence is 20 (18 from 2022), but the physiological development of the brain continues until the age of 30, and the psychological development of coping skills continues throughout an individual’s life [10]. It has been reported that AYA patients whose adolescence or young adulthood was interrupted by a diagnosis of cancer have a variety of concerns at each life stage, both during and after cancer treatment [33]. According to a pilot survey of Japanese AYA cancer patients and survivors, their most common concern was their own future, but for patients and survivors who were diagnosed at age 15–19 years, the second and the third most common concern was education (Table 2). Work and finance were the major concerns for patients aged 20–39 years. Late effect and fertility were also major concerns, especially for survivors after cancer treatment (Table 2).

**Table 2:** Major concerns of adolescents and young adults with cancer during and after treatment in Japan

Rank	Age 15–19 years	Age 20–24 years	Age 25–29 years	Age 30–39 years
Patients during cancer treatment (*N* = 213)				
1	Own future	Own future	Work	Own future
2	Education	Work	Own future	Work
3	Physical fitness	Finance	Finance	Finance
4	Diagnosis and treatment	Diagnosis and treatment	Fertility	Family
5	Late effect	Late effect	Diagnosis and treatment	Fertility
Survivors after cancer treatment (*N* = 132)				
1	Own future	Own future	Fertility	Own future
2	Late effect	Late effect	Own future	Work
3	Education	Fertility	Late effect	Fertility
4	Fertility	Work	Heredity of cancer	Physical fitness
5	Work	Marriage	Work	Late effect

Patients during cancer treatment were defined as those who were undergoing cancer treatment at the time of the survey or who had completed cancer treatment within 1 year. Survivors after cancer treatment were defined as those who were diagnosed with cancer between the ages of 15 and 39 and who had completed cancer treatment at least one year previously. Adapted from: Shimizu C., ‘The current situation and challenges of cancer patients in the AYA generation’. https://www.mhlw.go.jp/file/05-Shingikai-10901000-Kenkoukyoku-Soumuka/0000186548.pdf

Regarding completion of their education, according to a report from the pediatric patient experience survey 2019 conducted by the National Cancer Center in Japan, 61.3% of high school students took a leave of absence and 8.8% dropped out of school due to cancer treatment [34], compared with a drop-out rate of 1.3% among high school students in Japan as a whole [35]. The proportion of high school students with cancer who have been able to combine treatment and education was clearly lower than that of primary and secondary school students with cancer, who were in compulsory education and who were well supported by in-hospital classes [34].

Regarding continuing work, it has been reported that AYA cancer survivors are less likely to be employed than people without a history of cancer [36]. According to a report from the patient experience survey 2018 conducted by the National Cancer Center in Japan, 20.5% of patients aged 19–39 years became unemployed due to cancer treatment, while in the general population of 15–44 year-olds, the total unemployment rate was under 5% [37].

Financial issues for AYAs have been reported to be a barrier to care at specialized hospitals, and consequently contribute to poor outcomes [9, 38]. In Japan, in addition to the public medical insurance system under the universal coverage scheme, the Japanese government has subsidized medical expenses for children and adolescents (under 18 years of age) with cancer since 1974 [6] and since 2000 a public long-term care insurance system has been available for Japanese residents aged over 40 years. However, financial support for cancer patients aged 19–39 years is inadequate compared to the other age groups. Considering that this generation generally have lower incomes [39] and may need to bear their medical expenses themselves, to continue their academic or social career, or to care for their children or parents, governmental financial support might be important not only for children but also AYAs.

As AYA patients are of reproductive age, they may have sexual and reproductive health-related concerns and unmet needs that can affect future relationships, self-image, health, and quality of life [10]. Recommendations on fertility preservation in cancer patients were published in 2006 by the American Society of Clinical Oncology (ASCO) [40]. These included advice such as: “As part of education and informed consent before cancer therapy, oncologists should address the possibility of infertility with patients treated during their reproductive years and be prepared to discuss possible fertility preservation options or refer appropriate and interested patients to reproductive specialists” [40]. However, according to a report from the patient experience survey 2018 in Japan, only 52.0% of patients aged 19–39 years were informed of the fertility consequences of cancer treatment [41]. JSCO has published guidelines on the risk classification of infertility due to cancer treatment for Japanese oncologists [42]. The Japan Society for Fertility Preservation and regional networks for bridging oncology and reproductive medicine have also been established. In addition, a public subsidy system for fertility preservation in cancer patients was launched in 2021. It is hoped that all AYA patients will be informed about their infertility risk prior to treatment.

There are many other issues that should be addressed in the care of AYA patients, including hereditary cancer issues, survivorship and transition, and end-of-life care [28]. Peer support has been shown to be as important as support from family for this generation, and Japanese peer support organizations such as STAND UP!! have been established [43].

### The need for multidisciplinary care and cancer strategy in Japan

Both the clinical and psychosocial needs of AYA patients mandate a multidisciplinary approach to care with an extended group of medical, psychological, allied health care, social, and educational professionals [28].

The National Cancer Control Act in Japan was established in 2006, and based on the Basic Plan to Promote Cancer Control Programs (Table 3), a total of nearly 400 hospitals that meet the national criteria for the number of cancer patients, the quality of multidisciplinary staff, and availability of support programs for cancer patients have been designated as Core Cancer Treatment Hospitals by the Ministry of Health, Labour and Welfare (MHLW) [11, 44]. However, even among these designated cancer care hospitals, there were disparities in the availability of specialists with expertise in the care of the AYA generation [45]. In 2012, the Phase Two Basic Plan to Promote Cancer Control Programs raised the issue of care for children and fifteen hospitals were designated as childhood cancer care hospitals by MHLW. In 2018, the Phase Three Basic Plan to Promote Cancer Control Programs raised, for the first time, the issue of care for AYA patients and the need to promote a certain degree of centralization, with a focus on AYA cancer care. However, there are relatively large numbers of AYA patients compared to children with cancer, and this group have more diverse life stages, a greater variety of needs, and psychosocial difficulties, making it harder to determine the ideal cancer care system for them. In Europe, the centralization of AYA cancer patients is underway; in the UK, 17 principal treatment centers (PTC) for teenagers and young adults have been recognized as specialist expert hospitals for this generation. These have recently been demonstrated to improve clinical quality of life [46]. In France, to serve a population of 67 million, there are eight larger centres with full AYA units and a further five smaller AYA programs [28]. On the other hand, in Australia, local support models have been adopted in each jurisdiction by the Australian Youth Cancer Service, rather than a centralized model [29]. In Japan, although cancer care systems specific to the AYA generation have not yet been established, a research group funded by the MHLW in Japan has suggested that cancer care hospitals should create support teams for the AYA generation and establish a network system of support, including social support from local government in each region [47]. The AYA Oncology Alliance, established in 2018, is carrying out academic activities, educational activities, social awareness, and human resource development in the field of AYA cancer care in Japan.

**Table 3:** History of cancer control in Japan

1963	Subsidy for cancer research by Ministry of Health and Welfare started
1981	Cancer became the leading cause of death
1984	Comprehensive 10-year strategy for cancer control (~ 1993)
1994	New 10-year strategy to overcome cancer (~ 2003)
2004	The 3rd-term comprehensive 10-year strategy for cancer control (~ 2013)
2005 May	Headquarters of Cancer Control in Ministry of Health, Labour and Welfare (MHLW)
2005 August	Action plan 2005 for promotion of cancer control
2006 June	Cancer Control Act enacted
2007 April	Cancer Control Act implemented
2007 June	Basic plan to promote cancer control programs formulated
2009 July	Headquarters of 50% cancer screening rate (MHLW)
2012 June	Basic plan to promote cancer control programs revised (Phase 2)
2013 December	Cancer Registration Promotion Act was enacted
2014 March	Comprehensive 10-year strategy for cancer research formulated (~ 2023)
2015 June	Organization of cancer summit
2015 December	Formulation of “Acceleration plan for cancer control”
2016 January	Enforcement of Cancer Registration Promotion Act was implemented
2016 December	Amendment and implementation of a law to revise a part of the Cancer Control Act
2016 December	Organization of Cancer Genome Medical Forum 2016
2018 March	Basic plan to promote cancer control programs revised (Phase 3)

Adapted from: The editorial board of the cancer statistics in Japan. CANCER STATISTICS IN JAPAN 2021. https://ganjoho.jp/public/qa_links/report/statistics/2021_en.html

## Future direction

For oncologists caring for AYA patients today, it is important, first, to overcome barriers between departments, medical specialists, and societies to find ways to improve knowledge exchange in this field. For example, greater knowledge sharing could help professionals working on the challenging cases of adult patients with pediatric-type tumors or adolescents with adult-type cancers. This requires tremendous effort, and involves building bridges between institutions that are separate in organization and distance; difficulties are further amplified by the need to open protocols at multiple institutions [10]. Dissemination of educational materials for medical staffs, including e-learning modules and clinical guide handbooks, is also essential to address inequalities in quality of care and to increase awareness of the challenging aspects of AYA cancer care. Some of these materials have been already developed by the AYA Oncology Alliance [48]. On an individual level, we can continue to advocate for our AYA patients by seeking opportunities for trial enrollment, building collaborations with multidisciplinary oncologists and psychosocial colleagues, and ensuring that our patients are aware of all financial resources available to them [10].

## Abbreviations

ALL: Acute lymphoblastic leukemia
ASCO: American Society of Clinical Oncology
AYAs: Adolescents and young adults
CNS: Central nervous system
COG: Children’s Oncology Group
ESMO: European Society for Medical Oncology
SIOPE: European Society for Paediatric Oncology
ICD-10: International Classification of Diseases, 10th revision
JALSG: Japan Adult Leukemia Study Group
JCCG: Japan Children’s Cancer Group
JSCO: Japan Society of Clinical Oncology
MHLW: Ministry of Health, Labour and Welfare
MCIJ: Monitoring of Cancer Incidence in Japan
PTC: Principal treatment centers
SWOG: Southwest Oncology Group
SEER: Surveillance, Epidemiology, and End Results
